# Interlinked relationship between e-cigarette use and physical activity behaviour among Malaysian university students who use e-cigarettes: A cross-sectional study

**DOI:** 10.1371/journal.pone.0354336

**Published:** 2026-07-28

**Authors:** Chrisminder Dain, Lei Hum Wee, Yin How Wong, Yin Yin Ooi, Ching Sin Siau, Suzanna Awang Bono

**Affiliations:** 1 School of Biosciences, Faculty of Health & Medical Sciences, Taylor’s University, Subang Jaya, Selangor, Malaysia; 2 PETRONAS Research Sdn Bhd, PETRONAS, Bandar Baru Bangi, Selangor, Malaysia; 3 School of Medicine, Faculty of Health & Medical Sciences, Taylor’s University, Subang Jaya, Selangor, Malaysia; 4 Research Centre for Community Health (ReaCH), Faculty of Health Sciences, Universiti Kebangsaan Malaysia, Jalan Raja Muda Abdul Aziz, Kuala Lumpur, Malaysia; 5 Digital Health for Medical Advancements Impact Lab, Taylor’s University, Subang Jaya, Malaysia; 6 School of Social Sciences, Universiti Sains Malaysia, Gelugor, Penang, Malaysia; King Abdulaziz University Faculty of Medicine, SAUDI ARABIA

## Abstract

**Introduction:**

E-cigarette (EC) use is increasing among young adults in Malaysia. However, evidence on how EC-related behaviours and perceived physical activity barriers are associated with physical activity participation remains limited. This study examined the associations between EC-related behaviours and perceived physical activity barriers with physical activity status among Malaysian university students who use EC.

**Methods:**

A cross-sectional online survey was conducted between December 2023 and July 2024 across six Malaysian universities. Of 660 respondents, 564 met the age and International Physical Activity Questionnaire (IPAQ) criteria and were included in the analysis. Physical activity was classified as active and inactive. Exposures included EC use frequency, dual use, nicotine content, nicotine dependence, EC knowledge, motivations and perceptions for EC use, EC-related side effects, and perceived physical activity barrier items across personal, social and physical domains. Associations were analysed using chi-square tests and binary logistic regression, with odds ratios (ORs) and 95% confidence intervals.

**Results:**

Overall, 64.9% were classified as active. At the crude level, EC use frequency, nicotine content, and dual-use status were associated with physical activity. However, these variables were not retained in the multivariable model due to multicollinearity. Nicotine dependence emerged as one of the strongest behavioural correlates of physical inactivity, with higher dependence associated with greater odds of physical inactivity. Perceived barriers to physical activity, particularly personal and social barriers, demonstrated the strongest and most consistent associations, with substantially higher odds of inactivity across barrier levels. EC knowledge and perception variables were not independently associated after adjustment, although item-level patterns were observed. Reported EC related symptoms were mainly gastrointestinal, respiratory, and neurological but were not analysed in relation to physical activity.

**Conclusion:**

Physical activity among Malaysian university students who use EC is more consistently associated with nicotine dependence and perceived barriers, than with sociodemographic or knowledge variables. These findings suggest that interventions may benefit from addressing behavioural dependence and contextual constraints. Given the cross-sectional design, these results should be interpreted as hypothesis-generating.

**Implications:**

Physical activity among university students who use EC appears more closely associated with nicotine dependence and perceived barriers than with knowledge or sociodemographic factors. While some EC use patterns showed crude associations, nicotine dependence emerged as one of the strongest behavioural correlates of physical inactivity. Lower perceived barriers, particularly personal and social, were consistently linked to higher activity participation. These findings suggest that intervention strategies may benefit from addressing behavioural dependence alongside reducing personal and social barriers to physical activity, particularly factors related to motivation, self-confidence, and competing demands. Integrating accessible, context-specific physical activity opportunities within EC cessation or harm-reduction programmes may enhance engagement and promote healthier lifestyle behaviours among young adults.

## Introduction

Tobacco use remains one of the leading causes of preventable morbidity and mortality worldwide, accounting for more than 8 million deaths annually, including approximately 1.3 million deaths attributable to second-hand exposure [1]. Although the global prevalence of conventional cigarette (CC) smoking has declined over recent decades, the tobacco epidemic continues to impose a substantial health and economic burden with global costs estimated at USD 1.4 trillion annually [2,3]. In response to tightening tobacco control policies, the tobacco industry has diversified its product portfolio, particularly through the introduction and expansion of electronic nicotine delivery system (ENDS), including electronic cigarettes (EC).

EC are battery-operated devices that aerosolise a liquid typically containing nicotine, propylene glycol, flavourings, and other chemical compounds. Marketed as a “safer” or “smoke-free” alternative to CC, EC have gained widespread acceptance, especially among adolescents and young adults [4,5]. However, emerging evidence indicates that EC aerosols contain multiple toxic substances, including formaldehyde, chromium, nickel, acetaldehyde, and tobacco-specific nitrosamines [6], which exert cytotoxic and oxidative effects and are associated with adverse respiratory outcomes [7,8]. These findings raise concerns that EC use may perpetuate nicotine dependence rather than facilitate cessation, in addition to causing health problems.

In Malaysia, a shift in nicotine consumption patterns is increasingly evident. While CC smoking prevalence declined from 21.3% in 2019 to 19.0% in 2023, EC use has risen from 0.8% in 2011 to 5.0% in 2023, with higher uptake observed among adolescents and young adults [9–11]. Among university students, EC use prevalence has been reported to range between 8% and 40%, indicating a substantial behavioural health concern within this population [12–15]. This demographic represents a critical transitional stage characterised by increased autonomy, academic demands, and psychosocial stressors, all of which are associated with engagement in health-risk behaviours, including nicotine use and physical inactivity [16–18].

Physical inactivity is a major contributor to global disease burden and is associated with non-communicable diseases, poor mental health, and reduced functional capacity [19,20]. In Malaysia, the prevalence of physical inactivity increased from 25.1% in 2019 to 29.9% in 2023 [10,11]. Among university students, participation in physical activity is often constrained by barriers such as time limitations, competing academic priorities, and environmental factors [21–25]. Evidence from CC smoking populations consistently demonstrates an inverse relationship between nicotine dependence and physical activity, whereby individuals with higher dependence are less likely to engage in regular physical activity [26,27]. However, findings specific to EC users remain limited and inconsistent [28,29].

EC use behaviours are multifaceted, encompassing variations in use frequency, nicotine content, and levels of nicotine dependence. These characteristics may influence engagement in health-promoting behaviours such as physical activity through physiological, behavioural, and cognitive mechanisms [30–32]. For instance, higher nicotine dependence may reduce physical activity participation due to withdrawal symptoms, fatigue, or prioritisation of nicotine-seeking behaviour. In addition, contextual factors – such as perceived barriers to physical activity, knowledge regarding EC use, and perceptions of harm and benefit – may further shape behavioural outcomes [33–35].

Previous studies, both internationally and within Malaysia, have examined components of EC use and health behaviours, but often in isolation [13,22,24,36–41]. Malaysian studies have focused primarily on prevalence, patterns of EC use, and associated sociodemographic factors among university students [12–15], while separate lines of research have investigated physical activity levels and their barriers within similar populations [21–25]. For example, studies conducted across Malaysian universities have reported substantial variability in EC use prevalence and highlighted associations with sociodemographic and behavioural factors, although these studies did not examine physical activity concurrently [12–15]. Internationally, a consistent inverse relationship has been established between CC smoking and physical activity [26,27], however, evidence specific to EC use remains limited and inconsistent, with some studies suggesting differing behavioural patterns compared to traditional smokers [28,29]. Importantly, few studies have simultaneously examined EC use characteristics – such as frequency, nicotine content, and dependence – alongside contextual factors to understand their association with physical activity. This lack of integrated analysis limits understanding of how nicotine-related behaviours interact with lifestyle behaviours within young adult populations.

**Fig 1:**
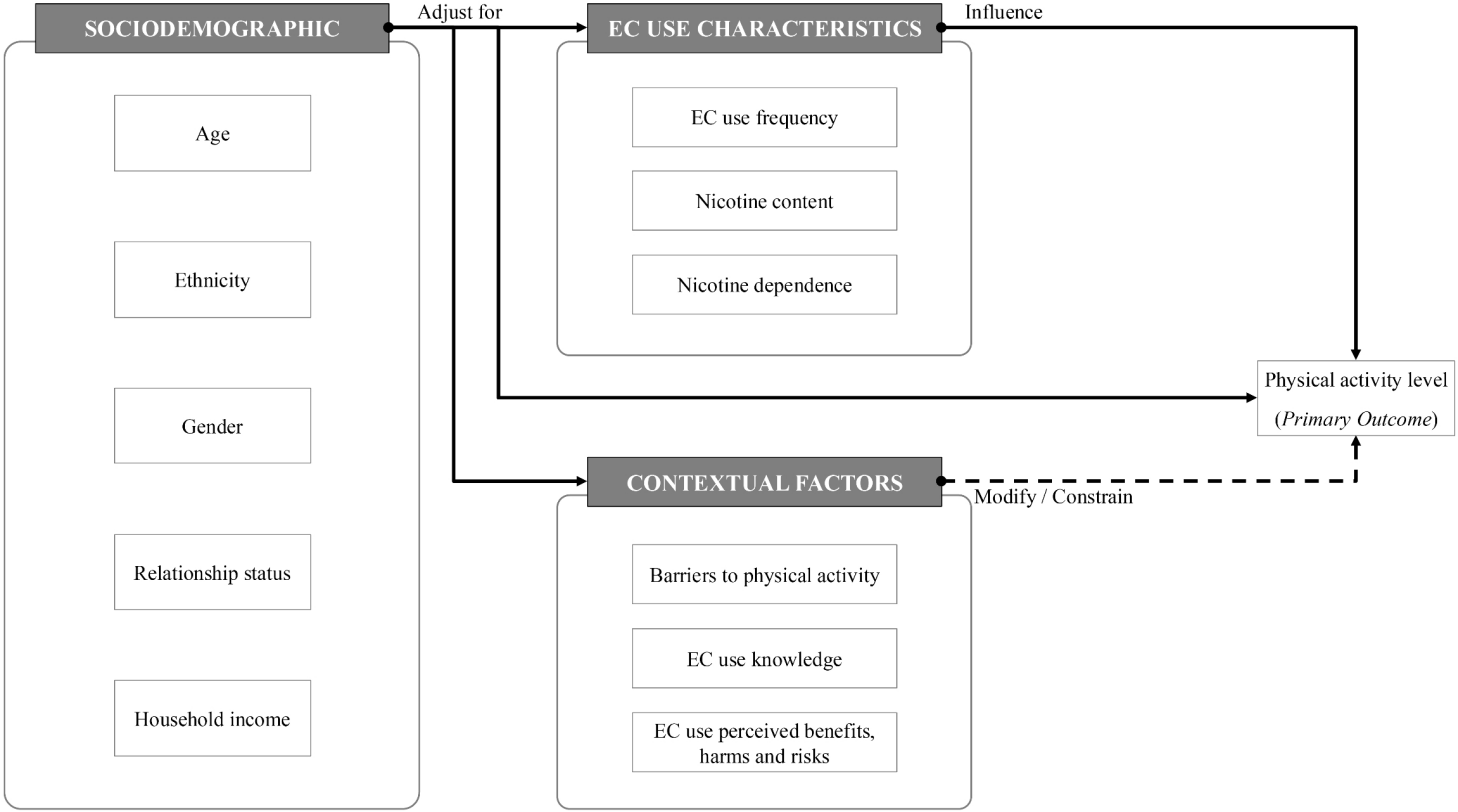
Research study conceptual framework.

To address this gap, the present study adopts a structured conceptual framework in which EC use characteristics (frequency of use, nicotine content, and nicotine dependence) are treated as primary exposures, physical activity level is specified as the primary outcome, and sociodemographic variables are included as background covariates. Contextual factors, including barriers to physical activity, EC-related knowledge, and perceived harms and benefits, are incorporated as modifying influences that may affect engagement in physical activity (Fig 1).

Accordingly, the primary objective of this study is to examine the association between EC use characteristics and physical activity level among Malaysian university students. Secondary analyses are conducted to characterise contextual and behavioural factors associated with physical activity; however, these are exploratory and intended to support interpretation of the primary association. This approach calls for more integrated behavioural analyses of EC use within Southeast Asian populations, where patterns of use and lifestyle behaviours may differ from Western contexts.

By explicitly prioritising physical activity as the primary outcome and situating EC use characteristics within a defined analytical framework, this study aims to provide a clearer understanding of the behavioural relationship between nicotine use and physical activity. The findings are expected to inform the development of targeted, non-pharmacological strategies to address the dual burden of EC use and physical inactivity among university students in Malaysia.

## Methods

### Study participants and settings

A cross-sectional survey was conducted between December 2023 and July 2024 across various universities in Malaysia. Data collection occurred at six sites – Site 1 (7 December 2023–8 January 2024), Site 2 (14 December 2023–11 July 2024), Site 3 (27 December 2023–4 April 2024), Site 4 (11 January 2024 and 13 June 2024), Site 5 (18 April 2024 and 30 May 2024), and Site 6 (19 June 2024 and 12 July 2024).

Initially, 10 institutions were targeted, however only six universities (three public and three private) agreed to participate. These institutions were strategically located across Peninsular Malaysia, with two in the northern region, one on the east coast region, two in the central region, and one in the southern region. This distribution ensures representation from diverse geographical and institutional contexts.

Due to the unavailability of complete enrollment lists, random sampling was not feasible which limited the possibility of probability-based recruitment. Consequently, a convenience sampling strategy was employed, allowing voluntary participation from students. While we recognise that this approach may introduce volunteer bias, it is important to note that the inclusion of both public and private institutions across multiple regions enhances sample diversity and its relevance to the broader student population.

To facilitate standardised recruitment, the lead researcher collaborated with the designated focal persons at each participating university. Students were approached through multiple channels, including email lists, classroom announcements, and social media platforms. These methods were standardised across all sites to ensure consistency in the recruitment process. Prior to accessing the survey, potential participants were verbally screened for eligibility criteria by the designated focal person. Only those who met the eligibility criteria proceeded to the full survey.

Ethical approval for the study was obtained primarily from Taylor’s University Human Ethics Committee (HEC 2023/265), Universiti Malaysia Kelantan Ethics Committee (UMK/PFV/HUMAN/EXT/0001/2024), and Universiti Teknologi MARA Ethics Committee (REC/03/2024). The remaining 3 universities agreed to use Taylor’s University ethics approval. To ensure transparent and rigorous research practices, informed consent was obtained electronically. The online survey platform was designed such that only participants who provided consent could proceed to complete the questionnaire. No personally identifying information was collected to maintain confidentiality and anonymity. These measures were implemented to ensure ethical integrity, participant protection, and methodological transparency. The standardisation of distribution procedures and consistent ethical safeguards also strengthen the reproducibility of the study’s approach and the generalisability of its findings across similar university populations.

Participants were eligible to participate in this study if they were (i) aged 18–25 years old, (ii) currently enrolled in a public or private university, (iii) exclusive EC users or dual users (defined as individuals who use both EC and CC concurrently) in the past month, and (iv) able to read and understand Bahasa Malaysia and English. Participants were excluded if they (i) had stopped using EC in the past month, (ii) were exclusive CC smokers only, (iii) were undergoing treatment for any psychiatric conditions, or (iv) refused to participate.

The study aimed to examine the relationship between EC use and physical activity to inform patterns of EC use and physical activity levels among university students who use ECs. Sample size was estimated using Cochran’s formula. Based on 65% quit motivation for EC users [12]; with a 95% confidence interval and a 5% significance level, it was determined that a minimum of 350 participants was required. Accounting for an anticipated 30% non-response or incomplete data rate, the target sample size was increased to 500. Although physical activity was the primary outcome of interest, reliable prevalence estimates for physically active EC users in Malaysian university populations were not available at the time of study planning. Therefore, quit motivation prevalence was used as the most appropriate and locally relevant behavioural parameter for sample size estimation.

In total, 660 EC users completed the survey. Following data cleaning, 37 cases were excluded for not meeting the age criteria, and a further 59 cases were excluded for not meeting the International Physical Activity Questionnaire (IPAQ) screening criteria, due to incomplete or improperly completed responses. For example, reporting participation in an activity (e.g., 3 days/week of vigorous activity) but leaving the corresponding duration (minutes/day) blank, reporting 0 days/week for an activity but also reporting a non-zero duration (e.g., 0 days of moderate intensity exercise but 45 minutes/day of moderate intensity exercise), and reporting extremely high frequencies and durations that result in implausible totals (e.g., 7 days/week of vigorous activity for 10 hours/day). This resulted in a final analytic sample of 564 participants.

### Study questionnaire

The cross-sectional study employed an online, self-administered questionnaire using TypeForm™, with an average completion time of about 20 minutes. The survey questionnaire was developed based on literature review, validated survey instruments and discussion among the research team members and comprised closed-ended questions organised into five main sections. The online survey platform was configured to require responses for all questionnaire items before participants could proceed to the next page or submit the survey. Although forced-response settings may reduce item non-response, it may also increase the likelihood of satisficing behaviours, such as selecting arbitrary responses to continue the survey. Nevertheless, previous methodological research suggests that forced-response formats can substantially reduce missing data, especially when questionnaires are concise, easy to complete, and relevant to respondents, with minimal impact on overall data quality [42].

### Sociodemographic characteristics

The first section captured sociodemographic characteristics (5 items) (i) age – (Input age), (ii) sex – (Male | Female), (iii) ethnicity – (Malay | Chinese | Indian | Other), (iv) relationship status – (Single | In a relationship | Married), and (v) monthly household income (MYR): < 2,499; 2,500−3,169; 3,170−3,969; 3,970−4,849; 4,850−5,879; 5,880−7,109; 7,110−8,699, 8,700−10,959; 10,960−15,039, > 15,039).

These categories were based on the Department of Statistics Malaysia (DOSM) framework, as used in national surveys such as the National Health and Morbidity Survey (NHMS) and the Household Income and Basic Amenities Survey (HIS/BA). For analysis, household income was further grouped into low income (< 4,850), middle income (4,850–10,959), and high income (> 10,959), in line with national reporting standards.

### Physical activity

The second section assessed physical activity using the International Physical Activity Questionnaire (IPAQ) [43]. Participants reported frequency and duration of vigorous activity, moderate activity, walking/cycling, and sedentary behaviours during a typical week (7 items). Activity levels were classified into: (i) low (Category 1; does not meet Category 2 and 3), (ii) moderate (Category 2; > 600 MET-min/week), and (iii) high (Category 3; > 3,000 MET-min/week). For this study, Category 1 was classified as “inactive”, while Categories 2 and 3 were classified as “active”.

### Barriers to physical activity

The third section assessed barriers to physical activity [21]. It consisted of 24 items or factors categorised into three domains (i) Personal (15 items), (ii) Physical Environment (5 items), and (iii) Social Environment (4 items). This questionnaire was adopted from a previous study on physical activity barriers conducted in Malaysia [21], which reported a Cronbach’s alpha value of 0.859, indicating good internal consistency. Items were rated on a five-point Likert scale ranging from “strongly disagree” to “strongly agree” (scored 1–5). Higher scores indicated greater perceived barriers and were categorised as low, moderate or high. For analysis, responses of “strongly disagree” and “disagree” were classified as “not a barrier”, while “agree” and “strongly agree” were classified as “a barrier”. Responses of “neutral” were analysed as a separate category.

### EC use and perceptions

The fourth section assessed EC use and related perceptions. The first sub-section characterised EC use profile using 5 indicators: (i) EC use frequency (everyday, a few times a week, a few times a month), (ii) nicotine content (with nicotine, without nicotine, unsure), (iii) nicotine concentration (< 10 mg, 10 mg - 19 mg, 20 mg - 40 mg, 41 mg - 60 mg, > 60 mg, unsure; adapted from a Malaysian study [13]), (iv) age of use initiation (input age), and (v) conventional cigarette use (yes / no). Participants reporting both EC and CC use were classified as dual users, while others were classified as exclusive EC users [13].

The second sub-section assessed reasons for EC use, where participants selected 1 out of 8 options: to stop smoking CC, to try EC, to reduce smoking CC, to feel cool and follow the latest trend, to fit into social groups, to reduce the cost, to use EC in a non-smoking area, or other [13].

A third sub-section assessed perceived EC-related side effects, where participants selected up to 5 symptoms from 18 options [13]. A total of 2,820 responses were generated from 564 participants. Although causality between EC use and symptoms cannot be established, the inclusion of this section improves ecological validity and contextual understanding of EC use patterns [38,44,45].

The final sub-sections assessed (i) EC knowledge (9 items), and (ii) the perceived benefits, harms and risk of EC use (13 items). The knowledge question was adapted from Baobaid et al. (2021) [36] with responses scored as “yes” or “no” (2 points and 1 point respectively). Items 7–9 were reversed scored. Internal consistency was acceptable (Cronbach’s alpha = 0.76). Higher scores indicated greater knowledge and were categorised as poor, fair or good. The perception questionnaire was adapted from Abdulrahman et al (2020) [46] and initially comprised of 10 items. Three additional items were added to assess reasons for use namely “own desire”, “social influence”, and “peer pressure”. Responses were recorded as either “yes” or “no”, and the questionnaire demonstrated good internal consistency (Cronbach’s alpha = 0.83).

### Nicotine dependence

Finally, nicotine dependence was assessed using the modified e-Fagerström test for nicotine dependence (e-FTND), which measures physical dependence on nicotine in EC users [47]. Internal consistency was acceptable (Cronbach’s alpha = 0.725). It contains 6 items assessing consumption patterns, compulsion to use, and overall dependence level.

### Data analysis

Statistical analysis was done using Microsoft Excel and IBM SPSS version 29.0. Descriptive statistics including frequencies (n), and percentages (%) were used to summarise categorical variables. Associations between variables were examined using chi-square (χ^2^) – specifically to determine which variable had significant associations with physical activity level. Binary logistic regression was performed with physical inactivity as the dependent variable (inactive = 1, active = 0) to identify factors independently associated with physical activity status by calculating odds ratio (crude odds ratio = cOR | adjusted odds ratio = aOR). Statistical significance was determined at an alpha (α) level of 0.05, and 95% confidence intervals (CI) were reported for effect estimates.

## Results

**Fig 2:**
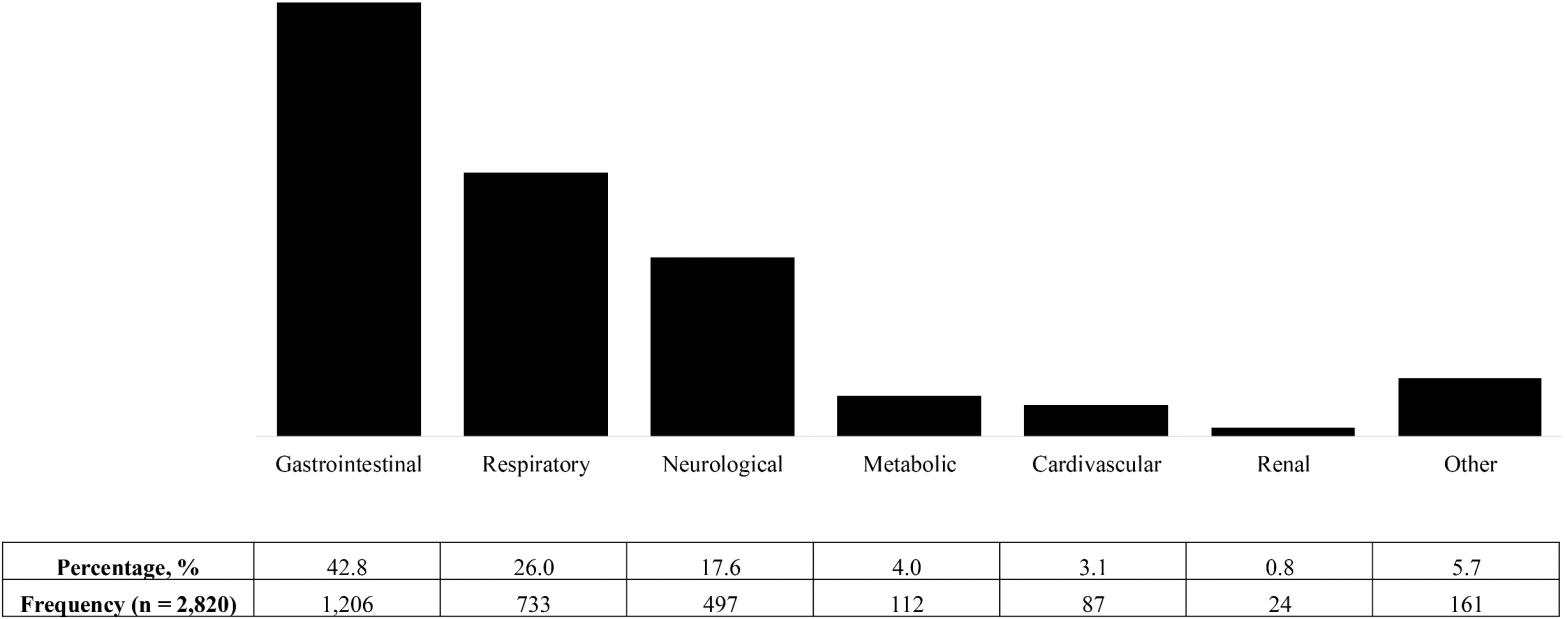
E-cigarette use potential side-effects.

The study included 564 Malaysian university students who reported current EC use. Overall, 64.9% (n = 366) were classified as physically active, while 35.1% (n = 198) were inactive based on IPAQ classification. Descriptive characteristics of the sample, including sociodemographic profile, EC use patterns, and physical activity levels and barriers, are presented in Table 1. Participants reason for EC use is summarised in Table 2, while perceived benefits, harms, and risk of EC use are detailed in Table 3. Associations between EC use characteristics, contextual factors and physical activity are examined using crude and adjusted ratios in Table 4, with item-level analyses of EC knowledge and physical activity barriers provided in S1 Table and S2 Table, respectively. Lastly, perceived side effects associated with EC use are illustrated in Fig 2.

**Table 1:** Sociodemographic, E-Cigarette Use Behaviour and Physical Activity (N = 564).

Variables	Frequency (n)	Percent (%)
**Age, years (Mean = 21.8 years old; SD = 1.69)**		
18–21	240	42.6
22–25	324	57.4
**Sex**		
Male	447	79.3
Female	117	20.7
**Ethnicity**		
Malay	375	66.5
Chinese	74	13.1
Indian	59	10.5
Other	56	9.9
**Relationship Status**		
Single	444	78.7
In a relationship	106	18.8
Married	14	2.5
**Monthly household income (MYR)**		
Less than 4,850	316	56.0
4,850–10,959	170	30.1
More than 10,959	78	13.8
**Electronic cigarette use frequency**		
Every day	428	75.9
A few times a week	95	16.8
A few times a month	41	7.3
**Age of electronic cigarette initiation, years (Mean = 18.1 years old; SD = 2.22)**		
10–15	82	14.5
16–20	425	75.4
21–25	57	10.1
**Nicotine dependence level (e-FTND)**		
High	181	32.1
Moderate	216	38.3
Low-to-Moderate	88	15.6
Low	79	14.0
**Electronic cigarette nicotine content**		
Without nicotine	241	42.7
Less than 10 mg	88	15.6
Between 10 mg and 19 mg	158	28.0
Between 20 mg and 40 mg	55	9.8
More than 40 mg	22	3.9
**Conventional cigarette use**		
Yes	234	41.5
No	330	58.5
**Electronic cigarette knowledge**		
Good	332	58.9
Fair	221	39.2
Poor	11	2.0
**Physical activity level**		
High	235	41.7
Moderate	131	23.2
Low	198	35.1
**Physical activity barrier**		
**Personal – lack of energy, lack of motivation, lack of self-confidence**		
High	71	12.6
Moderate	234	41.5
Low	259	45.9
**Social Environment – lack of support (from family, friends), lack of time**		
High	63	11.2
Moderate	264	46.8
Low	237	42.0
**Physical Environment – lack of facilities, lack of money**		
High	70	12.4
Moderate	293	52.0
Low	201	35.6

Note. e-FTND = e-Fagerström Test of Nicotine Dependence. MYR = Malaysian ringgit

**Table 2:** Reasons for Using E-Cigarette (N = 564).

Variables	Frequency			
	Yes		No	
	n	%	n	%
To stop smoking conventional cigarettes	170	30.1	394	69.9
To try electronic cigarettes	151	26.8	413	73.2
To reduce smoking conventional cigarettes	87	15.4	477	84.6
To feel cool and follow the latest trend	39	6.9	525	93.1
To fit into social groups (friends or colleagues)	25	4.4	539	95.6
To reduce the cost of smoking conventional cigarettes	19	3.4	545	96.6
To use electronic cigarettes in a non-smoking area	15	2.7	549	97.3
Other	58	10.3	506	89.7

**Table 3:** E-cigarette User Perceived Benefits, Harms and Risks (N = 564).

Variables	Frequency			
	Yes		No	
	n	%	n	%
I get definite nicotine hit from electronic cigarettes	402	71.3	162	28.7
Electronic cigarette use is as satisfying as conventional cigarette smoking	423	75.0	141	25.0
I like electronic cigarettes because it looks and feels like a conventional cigarette	341	60.5	223	39.5
Electronic cigarettes feel healthier than conventional cigarettes	399	70.7	165	29.3
Electronic cigarettes have helped me cut down smoking conventional cigarettes	439	77.8	125	22.2
I don’t have the urge to smoke conventional cigarettes as much since using electronic cigarettes	456	80.9	108	19.1
I crave for electronic cigarettes as much as I do for conventional cigarettes	381	67.6	183	32.4
I frequently use electronic cigarettes in places where smoking conventional cigarettes is banned	365	64.7	199	35.3
Electronic cigarettes have helped me to stop smoking conventional cigarettes	422	74.8	142	25.2
Electronic cigarettes allow me to use nicotine more	344	61.0	220	39.0
I use electronic cigarettes out of my own desire	459	81.4	105	18.6
I use electronic cigarettes because of social influence	349	61.9	215	38.1
I use electronic cigarettes because of peer pressure	295	52.3	269	47.7

**Table 4:** Crude and Adjusted Odds Ratio for Factors Associated with Physical Activity among E-cigarette users (N = 564). (Outcome: Physical Activity Level; Inactive = event category, Active = reference category).

Variables (*Reference group*)	cOR				aOR†			
	OR	95% CI		p	OR	95% CI		p
		LL	UL			LL	UL	
**Age group, years (*22–25*)**								
18–21	0.931	0.656	1.321	0.687				
**Sex (*Female*)**								
Male	1.052	0.686	1.615	0.815				
**Ethnicity (*Other*)**								
Malay	2.427	1.111	5.300	0.026	1.637	0.674	3.975	0.276
Chinese	9.857	4.073	23.853	<0.001	3.628	1.276	10.313	0.016
Indian	9.391	3.768	23.406	<0.001	4.319	1.476	12.633	0.008
**Relationship status (*Married*)**								
In a relationship	1.607	0.505	5.116	0.422				
Single	0.855	0.281	2.598	0.783				
**Monthly household Income (MYR) (*More than 10,959*)**								
4,850–10,959	2.041	1.178	3.536	0.011	1.710	0.815	3.585	0.156
Less than 4,850	0.535	0.316	0.906	0.020	1.129	0.544	2.342	0.745
**Electronic cigarette use frequency (*Monthly*)**								
Weekly	0.835	0.326	2.141	0.708				
Daily	2.826	1.275	6.264	0.011				
**Age of initiation, years (*21–25*)**								
10–15	1.615	0.770	3.390	0.205				
16–20	1.575	0.846	2.933	0.152				
**Nicotine Dependence Level, e-FTND (*Low*)**								
Low-to-Moderate	1.194	0.550	2.593	0.654				
Moderate	1.625	0.846	3.121	0.145				
High	7.193	3.755	13.779	<0.001	2.163	1.283	3.647	0.004
**Electronic cigarette nicotine content (*Without nicotine*)**								
Unsure	1.581	0.847	2.953	0.151				
≥ 20 mg	1.614	0.800	3.258	0.182				
< 20 mg	4.752	2.757	8.190	<0.001				
**Dual User (*Dual use*)**								
Exclusive electronic cigarette use	3.631	2.457	5.365	<0.001				
**Electronic cigarette knowledge (*Poor*)**								
Fair	1.086	0.322	3.664	0.894				
Good	0.433	0.129	1.454	0.175				
**Physical activity barrier – Personal (*Low*)**								
Moderate	4.324	2.801	6.675	<0.001	2.463	1.394	4.354	0.002
High	47.250	20.940	106.619	<0.001	22.253	6.774	73.104	<0.001
**Physical activity barrier – Social Environment (*Low*)**								
Moderate	4.108	2.680	6.296	<0.001	2.457	1.209	4.992	0.013
High	15.878	8.150	30.936	<0.001	1.898	0.568	6.342	0.298
**Physical activity barrier – Physical Environment (*Low*)**								
Moderate	6.875	3.777	12.513	<0.001	0.616	0.296	1.282	0.195
High	3.179	2.070	4.883	<0.001	0.498	0.175	1.420	0.192
**Main reason for using electronic cigarette (*No*)**								
To stop smoking conventional cigarettes	1.678	1.159	2.431	0.006	1.959	1.126	3.408	0.017
To try electronic cigarettes	1.419	0.967	2.083	0.074				
To reduce smoking conventional cigarettes	0.579	0.345	0.972	0.039	1.021	0.522	2.000	0.951
To feel cool and follow the latest trend	1.643	0.854	3.162	0.137				
To fit into social groups (friends or colleagues)	0.571	0.224	1.453	0.240				
To reduce the cost of smoking conventional cigarettes	0.482	0.158	1.474	0.201				
To use electronic cigarettes in a non-smoking area	0.454	0.127	1.628	0.225				
Other	0.308	0.148	0.641	0.002	0.668	0.280	1.591	0.362
**Electronic cigarette user perceived benefits, harms and risks (*No*)**								
I get definite nicotine hit from electronic cigarettes	2.064	1.367	3.116	<0.001	0.509	0.282	0.921	0.026
Electronic cigarette use is as satisfying as conventional cigarette smoking	2.438	1.558	3.815	<0.001	1.145	0.612	2.140	0.672
I like electronic cigarettes because it looks and feels like a conventional cigarette	1.847	1.28	2.664	0.001	0.726	0.405	1.301	0.282
Electronic cigarettes feel healthier than conventional cigarettes	1.729	1.159	2.578	0.007	0.883	0.504	1.548	0.664
Electronic cigarettes have helped me cut down smoking conventional cigarettes	1.313	0.856	2.014	0.212				
I don’t have the urge to smoke conventional cigarettes as much since using electronic cigarettes	1.698	1.061	2.718	0.027	1.058	0.542	2.065	0.869
I crave for electronic cigarettes as much as I do for conventional cigarettes	3.643	2.366	5.611	<0.001	1.440	0.791	2.622	0.233
I frequently use electronic cigarettes in places where smoking conventional cigarettes is banned	3.034	2.026	4.544	<0.001	0.931	0.521	1.661	0.808
Electronic cigarettes have helped me to stop smoking conventional cigarettes	1.671	1.098	2.545	0.017	0.675	0.365	1.249	0.211
Electronic cigarettes allow me to use nicotine more	3.989	2.658	5.985	<0.001	1.679	0.932	3.023	0.084
I use electronic cigarettes out of my own desire	3.37	1.941	5.853	<0.001	1.884	0.943	3.765	0.073
I use electronic cigarettes because of social influence	1.813	1.253	2.624	0.002	1.241	0.729	2.113	0.426
I like electronic cigarettes because it looks and feels like a conventional cigarette	1.387	0.979	1.965	0.066				

Notes. cOR = crude odds ratio. aOR = adjusted odds ratio. CI = confidence intervals. LL = lower limit. UL = Upper limit. MYR = Malaysian Ringgit. e-FTND = e-Fagerström Test of Nicotine Dependence. †Enter multiple logistic regression was applied. Hosmer-Lemeshow test (*p* = 0.088) and classification table (overall correctly classified percentage = 83.2%) were applied to check the model fit. Chi [2] (26) = 228.463, p < 0.001; Nagelkerke R [2] = 0.459. Electronic cigarettes use frequency, electronic cigarette nicotine content and dual user status were not entered into the multiple logistic regression model due to multicollinearity with nicotine dependence level.

### Sociodemographic characteristics and physical activity

Participants were aged between 18 and 25 years old (Mean = 21.8, *SD = ± 1.69*), with the majority aged between 22 and 25 years old, and the sample was predominantly single, male, Malay and monthly household income less than RM4,850 per month (Table 1). In inferential analysis (Table 4), age, sex, and relationship status were not associated with physical activity.

Ethnicity was associated with physical activity at the crude level (Table 4). Compared with participants in the “Other” ethnicity group, Malay participants had higher odds of being physically active (cOR = 2.43, 95%CI: 1.11–5.30), while Chinese and Indian participants showed even larger crude associations (cOR = 9.86, 95%CI: 4.07–23.85 | cOR = 9.39, 95%CI: 3.77–23.41, respectively). After adjustment, the association for Malay participants was attenuated and no longer statistically significant (aOR = 1.64, 95%CI: 0.67–3.98), whereas the associations for Chinese and Indian participants remained significant. Nonetheless, these findings should be interpreted cautiously because the reference category (“Other”) was relatively small (n = 56), which may contribute to unstable estimates and wide confidence intervals.

Household income showed a non-linear association with physical activity at the crude level. Compared with participants in the highest income category (> MYR 10,959), those in the middle-income group (MYR 4,850–10,959) had higher odds of being physically inactive (cOR = 2.04, 95%CI: 1.18–3.54), whereas participants in the lowest income group (<MYR 4,850) had lower odds of inactivity (cOR = 0.54, 95%CI: 0.32–0.91). However, these associations were attenuated after adjustment and were not statistically significant in the multivariable model (aOR = 1.71, 95%CI: 0.82–3.59 | aOR = 1.13, 95%CI: 0.54–2.34, respectively).

Overall, sociodemographic variables showed limited independent associations with physical activity, with significant effects observed primarily for specific ethnicity categories.

### E-cigarette use characteristics and physical activity

The initiation age of participants who use EC mainly ranged between 16 and 20 years old, with an average age of 18.1 years (*SD = ± 2.22*), and was not associated with physical activity.

In contrast, several EC use behaviours demonstrated associations at the crude level. Daily EC use was associated with higher odds of physical inactivity compared to monthly use (cOR = 2.83, 95%CI: 1.28–6.26). Similarly, exclusive EC users had higher odds of inactivity compared to dual users (cOR = 3.63, 95%CI: 2.46–5.37).

Nicotine dependence was one of the strongest and most consistent association. Compared against participants with low dependence, those with high dependence had substantially greater odds of physical inactivity (cOR = 7.19, 95%CI: 3.76–13.78), and this association remained significant after adjustment (aOR = 2.16, 95%CI: 1.28–3.65).

Nicotine content was associated with physical activity at the crude level, particularly for users of products containing less than 20 mg nicotine relative to nicotine-free products (cOR = 4.75, 95%CI: 2.76–8.19). However, this variable, along with EC use frequency and dual user status, was not included in the adjusted model due to multicollinearity with nicotine dependence.

Overall, EC use characteristics associated with physical activity at the crude level – including frequency of use, nicotine content, and dual-use status – appeared to reflect underlying nicotine dependence. Due to multicollinearity, these variables were not included in the multivariable model, where nicotine dependence emerged as the strongest EC-related behavioural correlate of physical activity.

### Reasons for EC use and physical activity

The most reported reasons for EC use were to stop smoking CC (30.1%), to try EC (26.8%), and to reduce smoking CC (15.4%) as shown in Table 2. In inferential analysis, using EC to stop smoking CC was independently associated with physical activity, with higher odds of physical inactivity (aOR = 1.96, 95%CI: 1.13–3.41). Although using EC to reduce smoking CC was associated with lower odds of inactivity at the crude level (cOR = 0.58, 95%CI: 0.35–0.97), this association was not retained after adjustment.

### Knowledge of EC use and physical activity

Overall, most participants have a good knowledge of EC (Table 1) but it was not associated with physical activity (Table 4). However, item level analysis (S1 Table) indicated that specific knowledge domains were associated with activity levels. Participants who were aware of EC regulations or perceived EC as less harmful or not harmful compared to CC demonstrated higher levels of physical activity. In contrast, general knowledge items – such as awareness of nicotine content, addictiveness, or chemical composition – were not associated with physical activity. These findings suggest that associations with physical activity are driven by specific cognitive and regulatory perceptions rather than overall EC knowledge.

### Perceived benefits, harms, and risks of EC use and physical activity

Participants reported high levels of perceived satisfaction, nicotine reinforcement, and smoking substitution associated with EC use (Table 3). The most frequently endorsed items were “used EC out of personal desire” (81.4%), “reduced urge to smoke CC” (80.9%), and “EC helping to cut down CC smoking” (77.8%), indicating strong perceptions of autonomy and substitution-related benefits.

At the crude level (Table 4), several perception-related variables were associated with physical activity, with many reflecting increased odds of physical inactivity among participants reporting stronger nicotine reinforcement and behavioural substitution effects (e.g., craving, satisfaction, and frequent use in restricted settings). However, most of these associations were attenuated after adjustment. The only perception-related factor that remained independently associated in the final model was reporting a definite nicotine hit from EC use (aOR = 0.51, 95%CI: 0.28–0.92), indicating lower odds of physical inactivity among those endorsing this perception.

Overall, these findings suggest that perception-related variables are closely aligned with behavioural patterns of EC use, and their crude associations with physical activity are largely accounted for by underlying nicotine dependence and related factors.

### Perceived side effects of EC use

Participants reported a range of perceived side effects associated with EC use (Fig 2). Across 2,820 total responses, gastrointestinal symptoms were the most frequently reported (42.8%, n = 1,206), followed by respiratory (26.0%, n = 733), and neurological symptoms (17.6%, n = 497). Less commonly reported were metabolic (4.0%, n = 112), cardiovascular (3.1%, n = 87), renal-related symptoms (0.8%, n = 24), and other symptoms (5.7%, n = 161).

Gastrointestinal = dry mouth, sore throat, bloating, nausea, stomach disturbances, vomiting. Respiratory = cough, breathing problem, asthma. Neurological = headache, anxiety. Metabolic = weight gain, diabetes. Cardiovascular = heart disease, high blood pressure, high cholesterol. Renal = kidney problem. Categories were referenced from Seiler-Ramadas et al, 2021 [38]

### Physical activity barriers

Perceived barriers to physical activity were assessed across personal, social environment, and physical environment domains (Table 1). Most participants reported low-to-moderate levels of barriers across all domains, with the largest proportions observed in the moderate category for physical environment (52.0%) and social environment barriers (46.8%), and in the low category for personal barriers (45.9%). High levels of barriers were less frequently reported, accounting for approximately 11% to 13% of participants across all domains.

In the adjusted model, personal barriers emerged as the strongest independent correlates of physical inactivity. Compared with participants reporting low personal barriers, those with moderate barriers had increased odds of inactivity (aOR = 2.46, 95%CI: 1.39–4.35), while those with high barriers had substantially greater odds (aOR = 22.25, 95%CI: 6.77–73.10). Moderate social barriers were also independently associated with inactivity (aOR = 2.46, 95%CI: 1.21–4.99), whereas high social barriers and physical environment barriers were not significant after adjustment. Although the magnitude of association for high personal barriers was strong and statistically significant, the relatively wide confidence interval suggests that the magnitude of the effect should be interpreted with caution.

Item-level analysis (S2 Table) showed that all 24 barrier items were associated with physical activity, with the strongest gradients observed for personal factors such as lack of motivation, embarrassment, perceived lack of benefit, lack of energy, and body image concerns.

In summary, these findings indicate that while certain sociodemographic differences persist, physical activity patterns among EC users are more strongly associated with behavioural factors – particularly nicotine dependence – and contextual constraints, especially personal and social barriers. The interplay between these factors provides a basis for interpreting how EC use behaviours and perceived barriers may jointly influence physical activity.

## Discussion

This study examined association between EC use characteristics, behavioural factors, and physical activity among Malaysian university students who use EC. Interpreted through the conceptual framework (Fig 1), the findings suggest that physical activity behaviour is shaped primarily through behavioural and contextual pathways – particularly nicotine dependence and perceived barriers – rather than by sociodemographic or knowledge factors alone. These associations remain correlational, given the cross-sectional design and the potential for reverse causation and residual confounding. Consequently, it cannot be determined whether higher nicotine dependence and greater perceived barriers contribute to lower physical activity participation, or whether lower levels of physical activity are associated with increased nicotine dependence and more pronounced perceived barriers. Therefore, the observed relationships should be interpreted as associations rather than evidence of causal pathways.

A key contribution of this study lies in positioning of EC use within a broader behavioural profile. The association between nicotine use and physical activity is not linear or uniform across populations. Prior evidence suggests that higher nicotine dependence is associated with lower engagement in physical activity, particularly when behavioural intensity is considered [31,32]. In contrast, studies that do not account for dependence often report mixed findings, including higher activity levels among EC users compared with non-users [48]. This inconsistency is reflected in the present findings, where nicotine dependence, rather than frequency of use, nicotine content or dual-use status, emerged as the primary EC-related correlate of physical inactivity. Other EC variables were excluded from the adjusted model due to multicollinearity. This supports the interpretation that these variables cluster around a common behavioural construct – namely dependence, rather than acting independently.

Although nicotine dependence emerged as an important behavioural correlate, the strongest associations observed in the multivariable model were related to perceived barriers to physical activity, particularly within the personal domain. Participants reporting high personal barriers were more than twenty times more likely to be physically inactive compared to those reporting low barriers. These barriers encompassed factors such as lack of motivation, low confidence, perceived lack of benefit, embarrassment, and limited energy for physical activity. These findings suggest that contextual and self-regulatory constraints may exert a greater influence on physical activity participation than EC use characteristics alone thus highlighting the importance of considering physical activity behaviour within a broader behavioural and environmental context rather than solely through nicotine-related pathways.

At the same time, the broader literature suggests that EC users do not represent a uniform risk group. For example, youth who use EC have been shown to be more physically active than traditional smokers, while still engaging in nicotine use [48]. Longitudinal evidence further complicates this relationship, showing that while higher physical activity may be associated with lower EC use cross-sectionally, yet may also predict future uptake, potentially due to perceptions of reduced harm [49]. Similarly, dual users and smokers are less likely to meet physical activity recommendations compared to EC-only users [28], while recent evidence suggests that EC use may reduce the likelihood of achieving high physical activity levels [34]. These findings indicate that the relationship between EC use and physical activity is heterogenous and context dependent, and is better understood when behavioural intensity and user profiles are considered.

Within this behavioural context, the present study provides further insight into the role of knowledge and perception. Although overall EC knowledge was high, it was not independently associated with physical activity. This aligns with studies showing that EC users often report higher subjective knowledge while simultaneously holding more favourable perceptions of EC use, including beliefs that it is safer, cost-effective, and useful for smoking cessation [36,37,50,51]. This suggests that knowledge may coexist with cognitive rationalisation, rather than directly promoting protective behaviour. In the present study, perception-related variables were attenuated after adjustment, supporting the interpretation that their influence is indirect and mediated through behavioural factors such as dependence and habitual use. Within the conceptual framework (Fig 1), knowledge and perceptions functions as intermediate constructs, influencing behaviour through more proximal mechanisms rather than acting independently.

An unexpected finding was that participants who reported obtaining a “definite nicotine hit” from EC use had lower odds of physical inactivity after adjustment. This result should be interpreted cautiously. The crude analysis demonstrated the opposite pattern, with perceived nicotine satisfaction associated with higher odds of inactivity, suggesting that the adjusted association may reflect the complex interrelationship between nicotine dependence, user perceptions, and behavioural characteristics rather than a direct protective effect of nicotine exposure itself. One possible explanation is that participants who perceive a clear nicotine effect may represent a subgroup of more experienced or intentional EC users who are nevertheless physically active and use ECs within recreational or social contexts rather than as highly dependent users. Previous studies have shown that EC users are a heterogeneous population, with some users maintaining active lifestyles despite ongoing nicotine use, particularly among younger adults and university students [28,48].

Alternatively, results of the multiple regression models may reflect a statistical suppression effect arising from simultaneous adjustment for nicotine dependence and multiple correlated perception variables. Perceived nicotine satisfaction is conceptually related to dependence, craving, satisfaction, and frequency of use, several of which demonstrated significant crude associations with physical inactivity. When highly correlated constructs are entered into the same multivariable model, coefficient reversal can occur, resulting in associations that differ substantially from unadjusted estimates. The reversal observed between crude (cOR = 2.06) and adjusted (aOR = 0.51) estimates is consistent with this possibility and suggests that the variable may be acting as a marker of a broader user profile rather than exerting an independent behavioural effect. Given the cross-sectional design and potential residual confounding, this finding should not be interpreted as evidence that nicotine reinforcement promotes physical activity. Rather, it highlights the complexity of the relationship between EC-related perceptions, dependence, and health behaviours, warranting further investigation using longitudinal designs and a more detailed measures of EC use patterns.

The Malaysian context further supports this interpretation. Wan Puteh et al (2018) [13] reported that EC use among Malaysian university students was strongly influenced by social norms, peer influence, and perceived benefits, including smoking cessation, self-image, and social engagement. Importantly, despite reporting symptoms such as dizziness, cough and headaches, many users did not perceive significant health risks. EC-related side effects were commonly reported across multiple physiological domains; however these variables were not analysed in relation to physical activity and therefore should not be interpreted as behavioural determinants in this study. Nonetheless, existing evidence indicates that EC use is associated with a broad range of symptoms, including respiratory, neurological, and gastrointestinal effects, likely mediated through inflammatory, oxidative, and neurophysiological pathways [38,52,53]. The current literature remains inconclusive, with predominantly cross-sectional evidence and substantial heterogeneity limiting causal inference. Within this context, EC use may be embedded within socially reinforced environment where perceived benefits, such as smoking substitution, nicotine satisfaction, and social acceptability outweigh perceived harms in shaping behaviour [13].

The strongest and most consistent findings in this study relate to perceived barriers to physical activity, which showed the largest and most robust associations. Within the conceptual framework, these barriers represent proximal determinants that directly constrain behaviour through limitations in motivation, time, energy, and competing priorities. This aligns with established behavioural models in which capability, opportunity, and motivation interact to determine behaviour [24,25,54].Systematic evidence indicates that environmental context, social influence, and goal prioritisation are among the most important domains influencing physical activity in university populations [54], while studies in Malaysia have similarly identified time constraints, academic demands, and motivational barriers as key determinants [21,55–57].

The present findings extend this evidence by demonstrating that personal barriers – particularly those related to motivation and perceived effort, are more strongly associated with inactivity than environmental barriers. This suggests that internal constraints may outweigh structural limitations in this population, even in contexts where access to facilities is available. EC use may indirectly reinforce these barriers. Wan Puteh et al (2018) [13] highlight that EC use is often linked with behavioural routines and coping mechanism, such as stress relief and social interaction, which may compete with engagement in physical activity. This supports the interpretation that the relationship between EC use and physical activity is not solely physiological but also behaviourally embedded in daily routines.

The interaction between EC use and physical activity may also be influenced by perceived health effects. Participants most frequently reported gastrointestinal, respiratory, and neurological symptoms, which may affect physical activity through discomfort or reduced exercise tolerance. These findings are consistent with previous evidence linking EC use to respiratory and systemic health effects [58,59] and with qualitative reports of perceived reductions in physical performance during physical activity among EC users [33]. Although self-reported, such experiences may discourage participation in physical activity and contribute to the gradual adoption of more sedentary behaviours over time.

Sociodemographic variables showed limited independent associations in this study, with only selected ethnic differences persisting after adjustment. This pattern is consistent with broader epidemiological findings in which demographic effects diminish once behavioural and contextual variables are considered [60,61]. The predominantly male sample reflects national patterns of EC use in Malaysia [11,62], but limits generalisability. Prior research suggests that female students may experience greater perceived barriers to physical activity, particularly in social and environmental domains, indicating that these associations may differ in more sex-balanced populations [63–65].

These findings support the conceptual framework (Fig 1) as a multi-layered behavioural system, in which EC use characteristics operate through behavioural mechanisms (nicotine dependence and routines), contextual constraints act as the strongest proximal determinants of physical activity, and knowledge and perceptions exert indirect influence, often mediated through perceived benefits and behavioural reinforcement. This integrated perspective suggests that physical activity among EC users is shaped by interaction between behavioural intensity, contextual constraints, and cognitive framing rather than isolated factors.

This study has several strengths, including a relatively large multi-university sample, validated instruments, and a theory-informed framework. However, several limitations should be acknowledged. The cross-sectional design limits causal inference and raises the possibility of reverse causation. Self-reported measures may introduce recall and social desirability bias. The lack of biomechanical verification of nicotine exposure limits interpretation of dose-response relationships, and unmeasured confounders such as mental health, academic stress, and sleep patterns may influence both EC use and physical activity. In addition, variability in EC products, usage patterns, and institutional environments may contribute to heterogeneity in observed associations.

Overall, the findings suggest that physical activity among EC users is shaped less by sociodemographic characteristics or knowledge and more by behavioural patterns (nicotine dependence and routines) and perceived environmental barriers. Future research should adopt longitudinal and mixed-methods approaches to better elucidate these pathways and to determine whether modifying dependence, perceptions, or contextual barriers leads to meaningful improvements in physical activity.

## Conclusion

In this cross-sectional study of Malaysian university students who use EC, physical activity patterns were more consistently associated with behavioural and contextual factors – particularly nicotine dependence and perceived personal barriers, than with sociodemographic characteristics or knowledge-related variables. Although participants generally demonstrated awareness of EC-related harms, this did not consistently correspond to more favourable physical activity patterns, suggesting that knowledge alone may be insufficient to influence behaviour in the presence of reinforcing perceptions and established routines.

From a practical standpoint, these findings indicate that interventions targeting university students who use EC may benefit from prioritising behavioural and contextual determinants, including strategies to address nicotine dependence and reduce key personal barriers such as motivation, time constraints, and competing academic or lifestyle demands. Approaches that incorporate behavioural support and context specific opportunities, such as accessible campus-based physical activity initiatives may be more effective than education-only strategies.

However, given the cross-sectional design and the potential for residual confounding and reverse causation, these implications should be interpreted as hypothesis-generating rather than prescriptive. Future longitudinal and intervention-based studies are needed to determine whether modifying nicotine dependence and contextual barriers can lead to sustained improvements in physical activity among EC users.

## Supporting information

S1 Table Item-Level Analysis of the Association between E-Cigarette Use Knowledge and Physical Activity (N = 564). (PDF)

S2 Table Item-Level Analysis of the Association between Physical Activity Barriers and Physical Activity (N = 564). (PDF)

S1 Data PLOS One Raw Data. (XLSX)

## Abbreviation:

CC: Conventional Cigarette
DOSM: Department of Statistics Malaysia
EC: Electronic Cigarette
e-FTND: Electronic Fagerström Test for Nicotine Dependence
ENDS: Electronic Nicotine Delivery System
HIS/BA: Household Income and Basic Amenities Survey
IPAQ: International Physical Activity Questionnaire
NHMS: National Health and Morbidity Survey
ReaCH: Research Centre for Community Health
WHO: World Health Organisation.
