# Supplementary material for: Interlinked relationship between e-cigarette use and physical activity behaviour among Malaysian university students who use e-cigarettes: A cross-sectional study
Source: PLoS One. 2026 Jul 28;21(7):e0354336. doi: 10.1371/journal.pone.0354336 (PMC13411885; doi:10.1371/journal.pone.0354336)
Supplement: S1 Table — (PDF) [file pone.0354336.s001.pdf]

Variables		Physical Activity Level (n, %)			Chi Square (df)	p-value
		Total (n)	Inactive	Active		
Electronic cigarettes are addictive	Yes	480	176 (*36.7*)	304 (*63.3*)	3.444 (1)	0.063
	No	84	22 (*26.2*)	62 (*73.8*)		
Electronic cigarettes have potential to cause asthma attacks and allergies	Yes	388	128 (*33.0*)	260 (*67.0*)	2.445 (1)	0.118
	No	176	70 (*39.8*)	106 (*60.2*)		
Electronic cigarettes can contain nicotine	Yes	510	179 (*35.1*)	331 (*64.9*)	0.000 (1)	0.990
	No	54	19 (*35.2*)	35 (*64.8*)		
Health risk of electronic cigarettes are the same as conventional cigarettes	Yes	344	126 (*36.6*)	218 (*63.4*)	0.896 (1)	0.344
	No	220	72 (*32.7*)	148 (*67.3*)		
Electronic cigarettes have the same chemicals as conventional cigarettes	Yes	238	74 (*31.1*)	164 (*68.9*)	2.912 (1)	0.088
	No	326	124 (*38.0*)	202 (*62.0*)		
Are you aware of any regulation by the government on electronic cigarettes?	Yes	316	75 (*23.7*)	241 (*76.3*)	40.796 (1)	<0.001
	No	248	123 (*49.6*)	125 (*50.4*)		
Electronic cigarettes are less harmful to health than conventional cigarettes	Yes	127	29 (*22.8*)	98 (*77.2*)	10.835 (1)	0.001
	No	437	169	268		

			(*38.7*)	(*61.3)*		
Electronic cigarettes are not harmful to health	Yes	326	97 (*29.8*)	229 (*70.2*)	9.712 (1)	0.002
	No	238	101 (*42.4*)	137 (*57.6*)		
Can electronic cigarettes be used at a smoke free place?	Yes	220	47 (*21.4*)	173 (*78.6*)	29.902 (1)	<0.001
	No	344	151 (*43.9*)	193 (*56.1*)		
