# Supplementary material for: Interlinked relationship between e-cigarette use and physical activity behaviour among Malaysian university students who use e-cigarettes: A cross-sectional study
Source: PLoS One. 2026 Jul 28;21(7):e0354336. doi: 10.1371/journal.pone.0354336 (PMC13411885; doi:10.1371/journal.pone.0354336)
Supplement: S2 Table — (PDF) [file pone.0354336.s002.pdf]

Variables		Physical Activity Level (n, %)			Chi Square (df)	p-value
		Total (n)	Inactive	Active		
**Personal**						
Item 1<br>I don't have extra energy to do physical activity after finishing university classes	Strongly Agree / Agree	126	61 (48.4)	65 (51.6)	86.215 (10)	<0.001
	Neutral	221	105 (47.5)	116 (52.5)		
	Disagree / Strongly Disagree	217	32 (14.7)	185 (85.3)		
Item 2<br>I feel sick and uncomfortable physically while exercising	Strongly Agree / Agree	92	66 (71.7)	26 (28.3)	123.373 (10)	<0.001
	Neutral	184	83 (45.1)	101 (54.9)		
	Disagree / Strongly Disagree	288	49 (17.0)	239 (83.0)		
Item 3<br>I have health problems which prevent me from being physically active	Strongly Agree / Agree	87	58 (66.7)	29 (33.3)	100.034 (10)	<0.001
	Neutral	169	84 (49.7)	85 (50.3)		
	Disagree / Strongly Disagree	308	56 (18.2)	252 (81.8)		
Item 4<br>Physical activity is difficult and tiring	Strongly Agree / Agree	125	68 (54.4)	57 (45.6)	72.470 (10)	<0.001
	Neutral	194	84 (43.3)	110 (56.7)		
	Disagree / Strongly Disagree	245	46 (18.8)	199 (81.2)		
Item 5<br>I look funny and feel ashamed when doing physical activities	Strongly Agree / Agree	121	79 (65.3)	42 (34.7)	132.758 (10)	<0.001
	Neutral	166	79	87		

			(47.6)	(52.4)		
	Disagree / Strongly Disagree	277	40 (14.4)	237 (85.6)		
Item 6<br>I am not interested in doing physical activities	Strongly Agree / Agree	96	68 (70.8)	28 (29.2)	122.876 (10)	<0.001
	Neutral	180	85 (47.2)	95 (52.8)		
	Disagree / Strongly Disagree	288	45 (15.6)	243 (84.4)		
Item 7<br>I don't get pleasure from physical activities or exercise	Strongly Agree / Agree	86	57 (66.3)	29 (33.7)	114.128 (10	<0.001
	Neutral	183	93 (50.8)	90 (49.2)		
	Disagree / Strongly Disagree	295	48 (16.3)	247 (83.7)		
Item 8<br>I think other recreational activities with friends and family members are more fun than exercise or physical activities	Strongly Agree / Agree	190	81 (42.6)	109 (57.4)	52.294 (10)	<0.001
	Neutral	226	93 (41.2)	133 (58.8)		
	Disagree / Strongly Disagree	148	24 (16.2)	124 (83.8)		
Item 9<br>I think physical activity is not beneficial to my health	Strongly Agree / Agree	75	60 (80.0)	15 (20.0)	132.111 (10)	<0.001
	Neutral	166	81 (48.8)	85 (51.2)		
	Disagree / Strongly Disagree	323	57 (17.6)	266 (82.4)		
Item 10<br>I am afraid of injury and fear for my safety when exercising	Strongly Agree / Agree	118	71 (60.2)	47 (39.8)	99.385 (10)	<0.001
	Neutral	201	89 (44.3)	112 (55.7)		

	Disagree / Strongly Disagree	245	38 (15.5)	207 (84.5)		
Item 11<br>I am too lazy to do physical activities	Strongly Agree / Agree	140	79 (56.4)	61 (43.6)	110.332 (10)	<0.001
	Neutral	200	91 (45.5)	109 (54.5)		
	Disagree / Strongly Disagree	224	28 (12.5)	196 (87.5)		
Item 12<br>Intensity of exercise required to get health benefits are too high for me	Strongly Agree / Agree	130	67 (51.5)	63 (48.5)	81.839 (10)	<0.001
	Neutral	213	99 (46.5)	114 (53.5)		
	Disagree / Strongly Disagree	221	32 (14.5)	189 (85.5)		
Item 13<br>I think I am not talented in doing physical activities	Strongly Agree / Agree	91	53 (58.2)	38 (41.8)	121.139 (10)	<0.001
	Neutral	193	102 (52.8)	91 (47.2)		
	Disagree / Strongly Disagree	280	43 (15.4)	237 (84.6)		
Item 14<br>I lack self-discipline / initiative in performing physical activities	Strongly Agree / Agree	189	87 (46.0)	102 (54.0)	72.700 (10)	<0.001
	Neutral	175	81 (46.3)	94 (53.7)		
	Disagree / Strongly Disagree	200	30 (15.0)	170 (85.0)		
Item15<br>My body shape doesn't allow me to do physical activities	Strongly Agree / Agree	90	57 (63.3)	33 (36.7)	139.143 (10)	<0.001
	Neutral	179	99 (55.3)	80 (44.7)		
	Disagree / Strongly Disagree	295	42 (14.2)	253 (85.8)		

**Social Environment**						
Item 16<br>My family members or friends don’t encourage me to do physical activities	Strongly Agree / Agree	95	66 (69.5)	29 (30.5)	114.656 (10)	<0.001
	Neutral	167	81 (48.5)	86 (51.5)		
	Disagree / Strongly Disagree	302	51 (16.9)	251 (83.1)		
Item 17<br>I don’t have friends to do physical activities together	Strongly Agree / Agree	140	74 (52.9)	66 (47.1)	91.470 (10)	<0.001
	Neutral	184	85 (46.2)	99 (53.8)		
	Disagree / Strongly Disagree	240	39 (16.3)	201 (83.8)		
Item 18<br>I don’t have free time to exercise or do physical activities because of my university workload	Strongly Agree / Agree	155	76 (49.0)	79 (51.0)	72.357 (10)	<0.001
	Neutral	195	90 (46.2)	105 (53.8)		
	Disagree / Strongly Disagree	214	32 (15.0)	182 (85.0)		
Item 19<br>I have to take care or help to take care of a dependent (such as siblings, parents)	Strongly Agree / Agree	93	47 (50.5)	46 (49.5)	75.268 (10)	<0.001
	Neutral	210	104 (49.5)	106 (50.5)		
	Disagree / Strongly Disagree	261	47 (18.0)	214 (82.0)		
**Physical Environment**						
Item 20<br>There are no facilities or places to do physical activities in my residential area	Strongly Agree / Agree	123	61 (49.6)	62 (50.4)	70.577 (10)	<0.001
	Neutral	181	90 (49.7)	91 (50.3)		
	Disagree / Strongly Disagree	260	47 (18.1)	213 (81.9)		

Item 21<br>Facilities or sports area are too far, and I don’t have any transportation	Strongly Agree / Agree	133	64 (48.1)	69 (51.9)	63.944 (10)	<0.001
	Neutral	187	90 (48.1)	97 (51.9)		
	Disagree / Strongly Disagree	244	44 (18.0)	200 (82.0)		
Item 22<br>I don’t know how to use sports equipment or specialties in doing physical activities	Strongly Agree / Agree	113	62 (54.9)	51 (45.1)	99.524 (10)	<0.001
	Neutral	194	96 (49.5)	98 (50.5)		
	Disagree / Strongly Disagree	257	40 (15.6)	217 (84.4)		
Item 23<br>The hot weather or rainy days prevent me to do physical activities	Strongly Agree / Agree	194	73 (37.6)	121 (62.4)	55.154 (10)	<0.001
	Neutral	205	98 (47.8)	107 (52.2)		
	Disagree / Strongly Disagree	165	27 (16.4)	138 (83.6)		
Item 24<br>I don’t have extra money to go to the sports facilities such as gymnasium or to buy sports equipment and clothes	Strongly Agree / Agree	178	71 (39.9)	107 (60.1)	65.837 (10)	<0.001
	Neutral	200	95 (47.5)	105 (52.5)		
	Disagree / Strongly Disagree	186	32 (17.2)	154 (82.8)		

Note 1: Categories “Disagree/Strongly Disagree” is classified as “Not a Barrier”

Note 2: Categories “Agree/Strongly Agree” is classified as “Barrier”
